# Renal Impairment as an Independent Predictor of Sepsis in Cirrhosis: A Retrospective Cohort Study

**DOI:** 10.3390/microorganisms14040785

**Published:** 2026-03-30

**Authors:** Mariana Boulos, Lana Majdoub, Maamoun Basheer, Nimer Assy

**Affiliations:** 1 Internal Medicine Department, Galilee Medical Centre, Nahariya 2210001, Israel; 2 Azrieli Bar-Ilan Faculty of Medicine, Bar-Ilan University, Safad 1311502, Israel

**Keywords:** sepsis, cirrhosis, laboratory analysis, ambulatory patients

## Abstract

Sepsis is a life-threatening complication among patients with liver cirrhosis and is associated with high morbidity and mortality. Early diagnosis is challenging due to immune dysfunction, chronic systemic inflammation, and overlap between clinical and laboratory findings during infection and hepatic decompensation. Therefore, there is a need to identify routinely available predictors that may enable the stratifying of patients at risk of developing sepsis in this population and facilitate intensive monitoring, antibiotic treatment, and potentially reduce mortality. The aim of this study is to evaluate the association between routine laboratory parameters and the development of sepsis among cirrhotic patients. A total of 171 cirrhotic patients met the inclusion criteria and were followed at a tertiary liver clinic between February 2015 and February 2022. Sepsis was defined according to Sepsis-3 criteria. Univariate analyses were performed to compare sepsis patients versus non-sepsis patients. Multivariable logistic regression was conducted to identify independent predictors of sepsis. Among 171 patients, 41 (24%) developed sepsis and 130 (76%) did not. Baseline characteristics were similar between groups: patients with sepsis were slightly older (67.5 ± 10.9 vs. 64.5 ± 12.3 years, *p* = 0.172), with no significant differences in sex (53.7% vs. 56.2%, *p* = 0.78) or ethnicity (Arab ethnicity 56.1% vs. 39.1%, *p* = 0.055). Ascites was more frequent in the sepsis group (53.7% vs. 26.2%, *p* = 0.001), whereas esophageal varices were less common (12.2% vs. 35.4%, *p* = 0.006). Rates of hepatic encephalopathy and acute kidney injury did not differ significantly. Higher creatinine (1.35 (0.80–3.35) vs. 0.80 (0.70–1.49) mg/dL, *p* < 0.001), INR (1.50 (1.20–1.80) vs. 1.30 (1.10–1.50), *p* = 0.011), and total bilirubin (1.90 (0.61–2.85) vs. 0.90 (0.59–1.70) mg/dL, *p* = 0.049) was observed in the sepsis group. In the multivariable model including age, sex, ethnicity, ascites, esophageal varices, INR, creatinine, neutrophil-to-lymphocyte ratio, and CRP, baseline serum creatinine was the only independent predictor of sepsis (adjusted OR 1.58 per 1 mg/dL increase, 95% CI 1.08–2.33, *p* = 0.01). Receiver operating characteristic (ROC) analysis demonstrated that the multivariable model had acceptable discriminative ability for prediction of sepsis, with an area under the curve (AUC) of 0.741 (95% CI 0.647–0.835). Among ambulatory patients with liver cirrhosis, baseline serum creatinine was independently associated with the development of sepsis. These findings highlight the need for dedicated risk-stratification tools in the outpatient setting. Further external validation in independent cohorts is required.

## 1. Introduction

Sepsis represents a major and life-threatening complication in patients with cirrhosis, contributing substantially to morbidity and mortality [1]. The clinical course of infection in this population is often atypical, and early diagnosis remains challenging due to altered immune responses and the overlap between manifestations of hepatic decompensation and systemic inflammation [2,3]. Current international guidelines emphasize the importance of rapid recognition and timely initiation of antibiotic therapy, as early intervention has been consistently associated with improved survival outcomes [4]. However, in cirrhotic patients, hepatic dysfunction may blunt classical signs of infection, thereby delaying diagnosis and treatment.

Patients with cirrhosis are susceptible to bacterial infections due to a complex immune dysregulation state characterized by persistent systemic inflammation and impaired host defense mechanisms [2,3]. This is due to a number of pathophysiological factors, including intestinal dysbiosis, increased gut permeability, and bacterial translocation, in addition to deficiencies in both innate and adaptive immune responses [5,6]. Furthermore, repeated hospitalizations, invasive diagnostic and therapeutic procedures, and the frequent use of indwelling catheters further elevate the risk of infection and subsequent sepsis in this population [1]. These mechanisms collectively create a unique clinical context in which infection can rapidly progress to organ dysfunction.

The diagnostic performance of standard sepsis criteria in cirrhosis has been questioned. Studies have demonstrated that conventional scoring systems may have limited sensitivity and specificity in patients with liver cirrhosis, emphasizing the need for more reliable diagnostic tools tailored to this population [7,8]. More recently, work by Anirban Dasgupta et al., published in Critical Care, highlighted the existence of a distinct inflammatory and clinical signature of sepsis in cirrhotic patients [9], further supporting the concept that traditional diagnostic markers may not fully capture early septic changes in this group.

Commonly used inflammatory biomarkers, such as C-reactive protein and procalcitonin, are frequently used in the diagnosis of sepsis; however, their interpretation in cirrhosis is complicated by impaired hepatic synthetic function and chronic baseline inflammation. These factors may result in altered biomarker kinetics and reduced diagnostic accuracy [10,11]. Studies have explored alternative inflammatory markers, including interleukin-6, yet no single biomarker has demonstrated sufficient reliability for widespread clinical implementation in cirrhotic populations [12,13]. Despite this, these biomarkers, especially CRP and procalcitonin, were primarily diagnostic for sepsis rather than predictive of future development [14,15,16,17]. Given these limitations, current research has increasingly focused on identifying accessible, cost-effective laboratory parameters that may facilitate earlier sepsis detection. Routine laboratory tests, which are widely available in clinical practice, may provide valuable insights into the inflammatory and coagulation pathways activated during infection. Platelet count, fibrinogen levels, and other hematological and biochemical markers have been shown to reflect systemic inflammatory responses and hepatic synthetic function, suggesting their potential utility as predictive indicators [12,13,18,19]. As a result, there is a clinically unmet gap that should be further explored.

In this regard, the present study aimed to evaluate the association between routine laboratory parameters and the development of sepsis in cirrhotic patients. Inflammatory and coagulation markers were systematically examined to determine their potential as accessible, clinically applicable predictors of sepsis onset.

## 2. Materials and Methods

### 2.1. Study Design and Setting

This retrospective cohort study was conducted at a tertiary hepatology referral centre. Patients were identified from the hepatology outpatient clinics between February 2015 and February 2022. This study aimed to evaluate clinical and laboratory predictors of sepsis among patients with cirrhosis during outpatient follow-up.

### 2.2. Study Population

Adult patients with a diagnosis of liver cirrhosis who were followed in the hepatology outpatient clinic during the study period were screened for eligibility using the institutional electronic medical records (EMRs). To ensure a consistent longitudinal observation framework, only patients who maintained regular hepatology clinic follow-up at approximately six-month intervals were eligible for inclusion. Baseline demographic and laboratory variables were obtained from the outpatient clinic visit preceding hospitalization and were retrospectively extracted from the EMRs.

The primary outcome was the occurrence of sepsis during hospitalization. For each eligible patient, the first hospitalization occurring within six months of an outpatient clinic visit was reviewed. Each patient contributed only one hospitalization to the study cohort. Potential sepsis cases were initially screened using ICD-9 discharge codes corresponding to sepsis, severe sepsis, or septic shock. Thereafter, all hospitalizations meeting the study inclusion criteria were manually reviewed by an internal medicine specialist, who extracted complete clinical and laboratory data from the electronic medical records for the first 24 h of admission. Baseline organ function was assumed to be normal (i.e., a baseline SOFA of zero) for the purpose of applying Sepsis-3 criteria. These cases were assessed, and patients were categorized into sepsis or non-sepsis groups. Hospitalizations without sepsis-related codes were likewise reviewed to confirm their classification as non-septic admissions. Importantly, based on patient or family member information, patients may have been hospitalized at other institutions; possible hospitalizations were screened using the national electronic health information system when available. However, due to ethical and data-access restrictions, detailed clinical information from hospitalizations occurring outside our institution was not retrieved. Therefore, patients whose first hospitalization occurred at another hospital were excluded from the analysis. A total of 171 patients met the study criteria and comprised the final analytic cohort.

### 2.3. Inclusion and Exclusion Criteria

Inclusion criteria were: (1) diagnosis of liver cirrhosis based on histological findings or on clinical assessment supported by biochemical markers, ultrasonography, endoscopic findings, or FibroScan; (2) age between 18 and 80 years; (3) referral and follow-up in our ambulatory hepatology clinic; and (4) compliance with regular clinic follow-up at approximately six-month intervals.

Exclusion criteria included: (1) lack of adherence to the six-month follow-up schedule; (2) hospitalization occurring at an external institution within the six-month follow-up interval; (3) hepatocellular carcinoma; (4) ongoing bacterial infection at baseline; (5) chronic kidney disease requiring dialysis; (6) overt hepatic encephalopathy; (7) severe extrahepatic disease, including congestive heart failure (NYHA class II or higher) or chronic obstructive pulmonary disease (COPD); (8) extrahepatic malignancy; (9) previous liver transplantation; (10) human immunodeficiency virus carrier; and (11) hospitalization at an external hospital.

Data collection and variables: Baseline demographic characteristics, cirrhosis etiology, cirrhosis-related complications, and medication use were extracted from the outpatient clinic visit preceding hospitalization. The documented etiologies of liver cirrhosis were hepatitis B virus (HBV), hepatitis C virus (HCV), alcohol-related liver disease, metabolic dysfunction-associated steatohepatitis (MASH), Wilson disease, autoimmune disease, primary biliary cholangitis, and primary sclerosing cholangitis. Comorbid conditions, including diabetes mellitus, hypertension, and chronic kidney disease, were documented. Liver disease severity was assessed using the Model for End-Stage Liver Disease (MELD), Chronic Liver Failure Consortium (CLIF-C-ACLF), and Fibrosis-4 (FIB-4) score [20] and Child–Turcotte–Pugh (CTP) scores, which were calculated for all patients [21,22,23]. CLIF-C-SOFA incorporates six organ systems (liver, kidney, brain, coagulation, circulation, and respiration). In our dataset, components were derived as follows: liver function was assessed using serum bilirubin, renal function using serum creatinine, coagulation using INR. We should emphasize that since vasopressor data were not available due to coding inconsistencies, maximal scoring points of hemodynamics was only 2 points. Furthermore, due to hepatic encephalopathy only being recorded as a binary variable (present vs. absent) without grading, encephalopathic patients received only 1 point regardless of the grading. Since respiratory parameters were not available, they were not included in the score calculation. Laboratory variables included complete blood count parameters (hemoglobin, white blood cell count, platelet count, and lymphocyte count), biochemical markers (serum creatinine, sodium, bilirubin, albumin, C-reactive protein (CRP), alanine aminotransferase (ALT), aspartate aminotransferase (AST), gamma-glutamyl transferase (GGT), alkaline phosphatase (ALP), and lactate dehydrogenase (LDH)), and coagulation profiles (prothrombin time (PT), partial thromboplastin time (PTT), fibrinogen, and international normalized ratio (INR)). D-dimer values were excluded from the analysis due to a high frequency of missing data, which precluded reliable statistical evaluation.

### 2.4. Outcome Definitions

Sepsis and septic shock were defined according to the Third International Consensus Definitions for Sepsis and Septic Shock (Sepsis-3). Sepsis was identified by a Sequential Organ Failure Assessment (SOFA) score ≥ 2. Septic shock was defined by the requirement for vasopressor therapy to maintain a mean arterial pressure (MAP) ≥ 65 mmHg in the presence of a serum lactate level ≥ 2 mmol/L [24].

### 2.5. Statistical Analysis

The categorical variables were summarized as frequencies and percentages. Continuous variables with a normal distribution were presented as mean ± standard deviation (SD), whereas non-normally distributed variables were expressed as median and range. Comparisons between categorical variables were performed using the Chi-square test or Fisher’s exact test. Continuous variables were compared between two groups using the independent-samples *t*-test for normally distributed data or the Mann–Whitney U test for non-normally distributed data. Variables associated with sepsis in the univariate analysis, as well as variables deemed clinically relevant, were analyzed by a multivariable logistic regression model to identify independent predictors of sepsis. A backward stepwise selection method was applied to derive the final model. A two-tailed *p*-value < 0.05 was considered statistically significant. Because the outcome was analyzed as a binary event (sepsis during hospitalization) and time-to-event data were not calculated, Cox proportional hazards regression was not applied. Multicollinearity among predictors was assessed using variance inflation factors (VIF). Model discrimination was evaluated using receiver operating characteristic (ROC) curve analysis and the area under the curve (AUC). Internal validation of the final multivariable model was performed using bootstrap resampling with 5000 iterations in the Rj module of Jamovi V 2.7.17.0 to estimate model optimism and derive optimism-corrected discrimination estimates.

Artificial intelligence-assisted tools were used to support certain aspects of manuscript preparation and literature exploration. Grammarly was used for language editing and text refinement. Literature screening and identification of relevant publications were facilitated using OpenEvidence and Elicit platforms. ChatGPT-5.4 was also used to assist with formatting tables derived from the results section and to provide general guidance on the statistical approaches used in this study. However, all study design decisions, data extraction, statistical analyses, and interpretation of results were performed by the investigators using SPSS 27.0.0 software and Jamovi.

## 3. Results

A total of 171 cirrhotic patients were included in the final analysis, in which 41 patients (24%) developed sepsis, while 130 patients (76%) did not. Patients who developed sepsis were slightly older (67.5 ± 10.9 vs. 64.5 ± 12.3, *p*-value = 0.172). Patients who developed sepsis had a higher proportion of Arab ethnicity compared with the non-sepsis group (56.1% vs. 39.1%), but this did not reach significance (*p*-value = 0.055). Comorbidities were also compared, without significant difference between the two groups. Beta blockers were observed in almost two-thirds of patients in the sepsis group and 75% in the non-sepsis group, with no significant difference (*p*-value = 0.298). Diuretics were used in approximately two-thirds of patients in both groups, but this difference was not significant. (Table 1).

The distribution of cirrhosis etiologies is presented in Table 2. Alcoholic-related cirrhosis had almost similar percentages among the no sepsis group and the sepsis group, 15.4% and 12.2% accordingly, without reaching statistical significance (*p*-value = 0.802). Metabolic-dysfunction-associated steatohepatitis (MASH) was the predominant etiology of cirrhosis in both groups (71 (54.6%) patients vs. 23 (56.1%) in the sepsis group), but still no statistically significant differences were observed among patients who developed sepsis and those who did not (*p*-value = 0.859).

Cirrhosis-related complications are shown in Table 3. In the non-sepsis group, 118 patients presented with acute decompensation, 90.7% compared to 40 (97.5%) in the sepsis group; this difference did not achieve statistical significance (*p*-value = 0.194). Ascites was significantly more prevalent among patients who developed sepsis (53.7% vs. 26.2%) in the non-sepsis group (*p*-value = 0.004). Counterintuitively, esophageal varices were significantly less common in the sepsis group (12.2%) compared with the non-sepsis group (35.4%, *p*-value = 0.006). Rates of hepatic encephalopathy and acute kidney injury did not differ significantly. Acute-on-chronic liver failure (ACLF) was markedly more frequent among patients with sepsis; 14 patients in the non-sepsis group had ACLF (10.8%) compared to 20 (48.8%) in the sepsis group, *p*-value < 0.001.

Markers of liver disease severity are summarized in Table 4. Median MELD-Na, Child–Turcotte–Pugh, and CLIF-C-ACLF and CLIF-C-SOFA scores were significantly higher among patients who developed sepsis, indicating more advanced liver disease at baseline.

Hematological laboratory parameters are presented in Table 5A. No significant differences were observed in hemoglobin, white blood cell count, or platelet count. In contrast, NLR was significantly higher in the sepsis group compared with the non-sepsis group (5.0 [3.0–8.5] vs. 3.0 [2.0–5.3], *p*-value = 0.002).

Biochemical and coagulation-related parameters are shown in Table 5B. Patients who developed sepsis had significantly higher serum creatinine levels 1.35 (0.8–3.35) than those without sepsis (0.8 (0.7–1.49). INR and total bilirubin levels were also significantly higher in the sepsis group. INR values had a median of 1.5 (1.2–1.8) in the sepsis group compared to 1.3 (1.1–1.5) in the non-sepsis group (*p*-value = 0.011). The median bilirubin in the sepsis group was 1.9 (0.61–2.85), and in the non-sepsis group this was 0.9 (0.59–1.7), which was significant with a *p*-value of 0.049. On the other hand, significant differences were not observed for sodium (*p*-value = 0.76) or fibrinogen (*p*-value = 0.14). Interestingly, CRP showed a trend toward higher values in the sepsis group but this did not reach statistical significance (*p*-value = 0.086). D-dimer analysis was excluded due to a high proportion of missing data.

Mortality was significantly higher among patients who developed sepsis compared with those who did not (48.8% vs. 12.3%, *p* < 0.001), as shown in Table 6.

The results of univariable logistic analysis are presented in Table 7A. Age and male sex were not associated with sepsis risk ((OR 1.02 per year, 95% CI [0.99–1.05], *p*-value = 0.172) and (OR 0.90, 95% CI [0.44–1.83], *p*-value = 0.771), respectively). Ethnicity, on the other hand, showed a non-significant association with sepsis risk. Jewish ethnicity was associated with lower odds of sepsis compared with Arab patients (OR 0.50, 95% CI [0.25–1.02], *p* = 0.057).

Among cirrhosis-related clinical variables, the presence of ascites was strongly associated with an increased risk of sepsis (OR 3.23, 95% CI [1.56–6.91], *p*-value = 0.002). In contrast, the presence of esophageal varices was associated with significantly lower odds of sepsis (OR 0.21, 95% CI [0.07–0.57], *p*-value = 0.002). Several laboratory parameters also demonstrated significant associations. Higher INR levels were associated with increased odds of sepsis (OR 2.06, 95% CI [1.08–3.91], *p* = 0.027). Baseline serum creatinine was strongly associated with sepsis development, with each 1 mg/dL increase in creatinine corresponding to a 76% increase in the odds of sepsis (OR 1.76, 95% CI [1.31–2.35], *p* < 0.001).

Neutrophil-to-lymphocyte ratio (NLR) was also associated with sepsis risk (OR 1.10, 95% CI [1.02–1.19], *p*-value = 0.015). Additionally, CRP demonstrated a statistically significant association with sepsis (*p*-value = 0.015). In contrast, serum sodium levels (OR 0.99, 95% CI [0.91–1.07], *p*-value = 0.758), platelet count (OR 1.00, 95% CI [0.96–1.00], *p*-value = 0.815), and fibrinogen levels (OR 1.00, 95% CI 0.98–1.00, *p* = 0.673) were not associated with the development of sepsis in univariable logistic analysis. Furthermore, as expected, several established liver disease severity scores were statistically associated with the development of sepsis. Higher MELD-Na scores were associated with sepsis (OR 1.08 per point increase, 95% CI [1.03–1.13], *p*-value < 0.001), as were higher Child–Turcotte–Pugh (CTP) scores (OR 1.42 per point increase, 95% CI [1.18–1.70], *p*-value < 0.001). The CLIF-C score also showed a statistically significant association (OR 1.05 per point increase, 95% CI [1.01–1.10], *p*-value = 0.008). However, the magnitude of MELD-Na and CLIF-C effects was modest, with odds ratios close to 1.

Results of multivariable logistic regression analysis are presented in Table 7B1. Variables that were statistically significant in the univariable logistic analysis were entered into the multivariable logistic regression model, along with key epidemiological variables considered potential confounders, including age, sex, and ethnicity. Severity scores were excluded due to substantial overlap with other significant parameters. After adjustment for age, gender, ethnicity, presence of ascites, varices, creatinine, INR, CRP, and NLR, baseline serum creatinine was the only variable independently associated with sepsis development (adjusted OR 1.58, 95% CI [1.08–2.33], *p* = 0.019). Other clinical variables, including age, sex, presence of ascites or esophageal varices, as well as inflammatory (CRP) and coagulation-related laboratory markers (INR), did not achieve statistical significance. For multivariable logistic analysis, after filtration of missing data, the patient number dropped to 164; the results are reported in Table 7B2. Creatinine remained significant with a minor decrease in odds ratio (OR = 1.55, 95% CI [1.06–2.19], *p*-value = 0.010).

**Figure 1:**
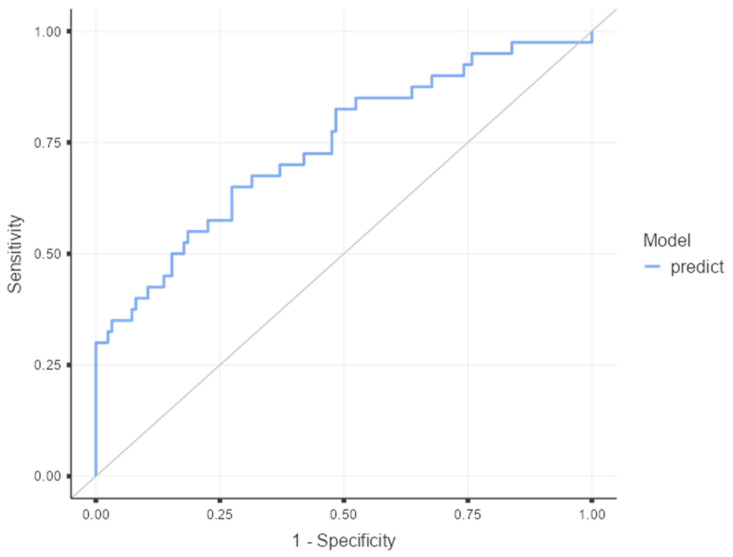
ROC curve of the multivariable prediction model for sepsis. Predicted probabilities were derived from the multivariable logistic regression model. The diagonal line represents the line of no discrimination (area under the curve = 0.5).

The discriminative performance of the multivariable model was assessed using ROC curve analysis (Figure 1), with the area under the curve (AUC) reported with 95% confidence intervals. The multivariable model demonstrated acceptable discriminative ability, with an area under the curve (AUC) of 0.741 (95% CI 0.647–0.835, *p* < 0.001).

**Figure 2:**
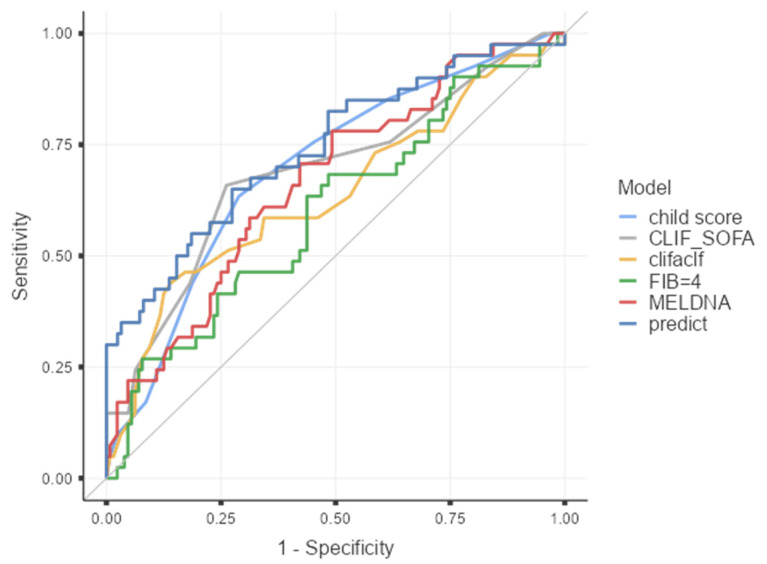
ROC curve comparison between the multivariable model and established liver severity scores. ROC curves were constructed to compare the predictive performance of the multivariable logistic regression model with MELD-Na, Child–Turcotte–Pugh (CTP), CLIF-C ACLF, CLIF-C-SOFA and FIB-4 scores. The analysis was performed in the complete-case cohort (*n* = 164). AUC values with 95% confidence intervals are reported in the ROC summary table. The diagonal line represents the reference AUC = 0.5).

To contextualize its performance, the model was compared with established liver disease severity scores. The multivariable model demonstrated superior discriminative ability compared with MELD-Na (AUC 0.666, 95% CI 0.571–0.761), Child–Turcotte–Pugh (AUC 0.698, 95% CI 0.606–0.789), and CLIF-C ACLF (AUC 0.637, 95% CI 0.532–0.743), and CLIF-C-SOFA modified showed discrimination (AUC 0.690, 95% CI 0.590–0.789). ROC curves for all models are presented in Figure 2.

Model calibration was assessed using the Hosmer–Lemeshow goodness-of-fit test, which demonstrated good agreement between predicted and observed probabilities (χ^2^ = 4.90, *p* = 0.769). Predicted and observed event rates across quartiles of predicted risk are shown in Table 8, further demonstrating the model’s adequate calibration (Table 9).

Internal validation of the multivariable logistic regression model was performed using bootstrap resampling with 5000 iterations (Table 10). The model’s apparent discrimination was good, with an AUC of 0.741. After correction for optimism, the internally validated AUC was 0.681. The bootstrap calibration slope was 0.713, suggesting moderate overfitting but acceptable calibration of the model predictions (Table 10).

To further explore the clinical relationship between renal dysfunction and sepsis, patients were stratified according to predefined creatinine categories (<1.0, 1.0–1.5, 1.5–2.0, and >2.0 mg/dL). Sepsis incidence was similar across lower categories (17–19%) but increased markedly in the highest category (48.6%), suggesting a threshold effect at higher levels of renal dysfunction (*p* < 0.001), Table 11.

## 4. Discussion

Our study included 171 ambulatory cirrhotic patients who were followed at a tertiary liver clinic. Sepsis developed in 41 patients (24%) during 2-year follow-up and was associated with a marked increase in mortality (48.8% vs. 12.3%, *p* < 0.001). Baseline creatinine was the only laboratory variable independently associated with significant sepsis development, whereas inflammatory and coagulation-related laboratory variables including CRP, platelet count, fibrinogen, did not demonstrate independent predictive value.

Our study’s observed sepsis incidence (24%) is consistent with the published rates among hospitalized cirrhotic patients (30–40% on hospital admission are complicated with bacterial infections). Multiple factors are involved in why patients with cirrhosis have substantially higher rates of bacterial infection and sepsis. Cirrhosis-associated-immune dysfunction (CAID) refers to immunodeficiency, immune function abnormalities, and chronic systemic inflammation that are present in cirrhosis [26]. It constitutes the hallmark of the greatest susceptibility to bacterial infection in cirrhotic patients. Infections, particularly spontaneous bacterial peritonitis (SBP), are the most common precipitant of acute decompensation and ACLF, which, as a result, increase mortality rates beyond those of non-cirrhotic sepsis [27,28]. These reported percentages are consistent with our study results of higher mortality rates among those with septic cirrhosis, 48% versus 12.3%, though we included outpatients only. Immuno-dysregulations, the difficulties in triggering inflammatory markers, and the tendency for higher organ failure rates (especially renal and hepatic) account for the higher death rates [1,9].

Unsurprisingly, septic patients had significantly higher baseline liver severity scores (MELD-Na and CLIF-C-ACLF; mean of 17.8 (13.6–24.7) vs. 13.3 (10.0–19.1), *p* < 0.001, and mean of 45 (37.5–52) vs. 40 (135–45), *p* = 006, respectively), affirming the fact that advanced cirrhosis confers more susceptibility for sepsis due to CAID. MELD-Na is a mortality-risk-quantifying tool that includes sodium levels, creatinine as a renal biomarker, the coagulable state biomarker INR, and bilirubin as a liver injury biomarker [29]. The chronic liver failure consortium acute decompensation (CLIF-C-AD) score is a survival-prognostic tool in acute decompensated cirrhosis; it also serves as a risk stratification tool to identify high-risk patients. It includes age, sodium, WBC, creatinine and INR [30]. The findings presented in our study may be explained by the fact that higher scores reflect more advanced liver damage and thus advanced dysregulation of immunity systems. Moreover, different studies have suggested more biological mechanisms that may be responsible for the increased vulnerability to sepsis: intestinal ascites is induced by significant portal hypertension and as a result bacterial translocation; pathogen associated molecular patterns (PAMPs) also translocate from the gut to circulation, triggering systemic inflammation. Constant activation of PAMPs results in additional immune dysfunction [31]. It is important to note that these scores chiefly reflect the body’s physiological reserve and general vulnerability, rather than providing insight into infection-specific biology, Therefore, although severity score strongly stratifies risk at the population level, it may not fully be representative of the specific mechanisms by which patients progress from infection to sepsis.

Among cirrhosis complications, ascites was significantly more prevalent among patients who developed sepsis (53.7% vs. 26.2%, *p* = 0.001). This finding is biologically feasible and clinically consistent with the portal-hypertension phenotype of decompensated cirrhosis due to a more complex disease. It is also linked to impaired gut barrier integrity and altered intestinal microbiota, thereby increasing exposure to bacterial proteins and prompting the systemic inflammatory response [32]. In advanced cirrhosis, significant splanchnic vasodilation and, as a result, systemic hypotension trigger neurohumoral vasoconstrictor systems—including the renin–angiotensin–aldosterone system, the sympathetic nervous system, and vasopressin release—which retain sodium and water, leading to plasma volume expansion and intense vasoconstriction in the renal circulation. This cascade impairs effective perfusion of vital organs, leading to organ dysfunction, especially hepatorenal syndrome, and predisposes to organ dysfunction, notably hepatorenal syndrome [33].

The lower prevalence of esophageal varices among patients in the sepsis group was an unexpected finding in this cohort, observed in multivariate analyses. The presence of esophageal varices was less associated with the sepsis group compared to the non-sepsis group (12.2% vs. 35.4%, *p*-value = 0.006), consistent with lower odds of sepsis development with OR = 0.82 (0.10–1.15), but did not reach statistical significance (*p*-value = 0.126). This is contradictory to our understanding, especially of the effects of significant portal hypertension discussed above; thus, it is unlikely to reflect a true protective effect of varices themselves. Instead, we believe it may represent clinical management patterns. Patients with documented varices usually undergo structured hepatologist follow-up, which results in more frequent clinical contact, earlier recognition of possible deterioration, and intensive intervention before progression to a septic state [34]. What is more, our data lack possible important confounders that might really affect our reported odds ratio and statistical significance. It must include the following: NSBB dosage and response rate, as studies reported up to 69% response rate among compliant patients [35], patient compliance [36,37], lifestyle modifications, and the severity of portal hypertension, which were not reported throughout the data and would, in our opinion, change the odds of the association. Not only that, but higher-risk patients are treated with non-selective beta-blockers (NSBBs). Independent of reducing portal hypertension, these drugs modulate immunity by reducing systemic inflammation (reducing WBC, CRP, interleukin-6 (IL-6), and procalcitonin), thereby decreasing bacterial translocation and the risk of bacterial infection [38,39]. We suggest that this finding should therefore be interpreted cautiously and warrants confirmation in larger prospective cohorts.

The neutrophil-to-lymphocyte ratio was significantly higher in patients who developed sepsis on univariate analysis (median of 5 (3–8.5) in the sepsis group, 3 (2–5.3) in the non-sepsis group, *p* = 0.002) but still did not retain significance in the multivariable model. The results in our study are consistent with the latest literature; one large critical-care database reported that higher NLR predicted mortality with moderate discrimination (area under the curve (AUC) = 0.75) [40]. Others have established this ratio as an independent value of MELD and CTP scores [41,42]. A meta-analysis by Lin et al. found mixed results, with some datasets showing limited prognostic association [43]. While most studies report results like those of this study, Salciccioli et al. found that NLR was not significantly different among septic patients, including 5056 patients [44]. Still, none of these studies examined the role of NLR in predicting sepsis development. NLR is a peripheral simple ratio that describes two parts of the immune system. The adaptive response is supported by lymphocytes, and the innate response is driven mainly by neutrophils [45]. Higher neutrophils in cirrhotic patients are due to the persistent innate immune system activation in response to PAMPs from bacterial translocation [46] and damage-associated molecular patterns (DAMPs) from hepatocyte injury [47]. This cascade stimulates pro-inflammatory cytokines that promote neutrophil mobilization into the peripheral circulation [48]. While in a septic state, blood lymphocytes increase in association with cell activation. Chronic systemic inflammation continuously stimulates lymphocytes, which eventually leads to exhaustion and decreased peripheral lymphocyte levels [49]. Thus, higher NLR is associated with but does not predict sepsis in cirrhotic patients.

The most important result of this study is that baseline creatinine was the only independent predictor of sepsis development (adjusted OR 1.58, 95% CI 1.08–2.33, *p* = 0.019). It is a laboratory biomarker of renal dysfunction. This finding might be explained by it acting not only as an isolated organ impairment, but also as a systemic marker reflecting immune dysregulation, endothelial dysfunction, circulatory failure, and diminished physiologic reserve [33]. Endothelial dysfunction, triggered by bacterial translocation and increased nitric oxide levels, causes splanchnic vasodilation and vascular tone. Portal hypertension exacerbates the splanchnic vasodilatory effect, leading to reduced effective volume and a hyperperfused kidney. To add more, In addition, septic cirrhosis dysregulated endothelial–coagulation interactions, as levels of anithrombin-2, soluble thrombomodulin, and von Willebrand factor are elevated in these patients [9,50,51]. Moreover, systemic inflammatory response syndrome (SIRS) is a frequent complication of cirrhotic patients, but it also leads to increased mortality [52,53]. It arises from the same immune dysregulation and persistent activation due to bacterial translocation, leading to a systemic inflammatory state that releases pro-inflammatory cytokines (IL-6 for example), triggering disease progression and organ dysfunction. This primarily occurs through increased vascular permeability, which may trigger microvascular thrombosis and, eventually, increased serum creatinine and reduced glomerular filtration [54,55]. In cirrhotic sepsis, the most precise and earliest clinical deterioration may occur through hemodynamic collapse and microvascular organ injury rather than through an excessive inflammatory biomarker rise.

Our multivariable model showed acceptable discrimination for sepsis prediction in cirrhosis, with an AUC of 0.741, and it outperformed the conventional liver severity scores assessed in our cohort, including MELD-Na (0.666), CTP (0.698), CLIF-SOFA (0.690), and CLIF-ACLF (0.637). This finding is clinically relevant because most of the contemporary high-impact literature on infection in cirrhosis has focused on hospitalized patients with acute decompensation or ACLF rather than ambulatory populations, leaving outpatient sepsis-risk stratification comparatively underexplored [6]. A reasonable explanation for the better performance of our model is that it incorporates variables more proximal to the biology of infection-related decompensation, particularly renal dysfunction and systemic inflammatory activity, whereas MELD-Na, CTP, CLIF-SOFA, and CLIF-ACLF were primarily designed to stage liver dysfunction, organ failure, or short-term prognosis rather than to predict incident sepsis itself [56,57].

Our findings are also directionally consistent with recent studies showing that conventional liver severity tools provide only moderate discrimination when applied outside the exact context for which they were developed. In a recent gut study of acutely decompensated cirrhosis, the MELD-Na score achieved an AUC of 0.787 for severe clinical trajectory, while a dedicated CLIF, systemic inflammation gene (CLIF-SIG), performed better (AUC 0.811), underscoring that models enriched with pathobiologically relevant features can outperform traditional severity scores [58]. Still, that study was directed toward prognosis rather that diagnostic performances. Importantly, we should spotlight that comparison with published data should be interpreted cautiously because most recent comparator studies in top-tier journals evaluated inpatient cohorts with acute decompensation, ACLF, or short-term mortality rather than incident sepsis in ambulatory cirrhosis.

In our study, missing respiratory data and simplified encephalopathy coding could have reduced the reliability of modified-CLIF-domain capture and therefore attenuated discrimination through non-differential misclassification [56]. Several sources of bias could also have influenced model performance. Missing data can introduce selection bias when complete-case analyses are required (our cohort dropped to 164 patients rather than the original 171), especially if unavailable laboratory values are related to illness severity, and outcome misclassification can occur when predictor domains are incompletely captured [57].

Clinically, our findings have several implications. First, they support our suggestion that outpatient renal dysfunction should be treated as a major warning marker for subsequent sepsis susceptibility. This biomarker is already part of routine cirrhosis care, is inexpensive, and is widely available. Second, the findings confirm that many commonly used biomarkers are not specific enough for cirrhosis and should not be the only biomarkers for excluding infection risk. Thirdly, given the high mortality associated with sepsis, earlier evaluation and lower thresholds for escalation of treatment may be warranted in patients with underlying renal insufficiency, particularly in the event of mild clinical deterioration.

This study has several strengths. It focuses on an ambulatory cirrhotic cohort, which is clinically important and less frequently studied compared to inpatients, especially ICU populations. It improves real-world applicability by assessing standard laboratory parameters. The use of Sepsis-3 criteria provides alignment with updated definitions. However, several limitations should be acknowledged alongside the strengths discussed above. The retrospective design introduces the risk of residual bias and misclassification bias, as well as data completeness issues. A single-centre setting may limit generalizability to other populations. The small study population may limit the power to detect modest independent associations with other biomarkers, which may explain why variables like NLR and CRP did not emerge as independent predictors in the regression model (a potential type 2 error). Another limitation would be, in our opinion, some unmeasured variables such as the severity of ascites, the degree of portal hypertension (hepatic venous pressure gradient (HVPG)) and beta-blocker dosage, as these could influence outcomes.

In conclusion, sepsis occurred frequently in ambulatory patients with cirrhosis and was associated with markedly increased mortality. Baseline serum creatinine was the only routine laboratory parameter independently predictive of subsequent sepsis development. These findings reinforce the unique phenotype of cirrhotic sepsis and support including renal dysfunction as a key aspect of ambulatory care and risk stratification. Taken together, these data suggest that a sepsis-oriented multivariable model may add clinically relevant information beyond conventional liver severity scores in ambulatory cirrhosis, but the present results should be viewed as hypothesis-generating until externally validated in independent outpatient cohorts with standardized infection adjudication and more complete organ-failure phenotyping.

## Figures and Tables

**Table 1:** Baseline demographic and clinical characteristics.

Variable	No Sepsis (*n* = 130)	Sepsis (*n* = 41)	*p*-Value *
Age, years (mean ± SD)	64.5 ± 12.3	67.5 ± 10.9	0.172
Male sex, *n* (%)	73 (56.2)	22 (53.7)	0.78
Arab ethnicity, *n* (%)	50 (39.1)	25 (56.1)	0.055
BMI, kg/m^2^ (mean ± SD)	28.9 ± 5.99	28.4 ± 5.44	0.655
Diabetes mellitus type 2, *n* (%)	83 (64.3)	28 (68.3)	0.64
Proton pump inhibitor use, *n* (%)	64 (49.6)	14 (35.0)	0.146
Beta-blocker use, *n* (%)	98 (75.4)	25 (65.8)	0.298
Statin use, *n* (%)	32 (24.6)	7 (17.1)	0.396
Diuretic use, *n* (%)	85 (68.5)	26 (63.4)	0.912

BMI: body mass index. Values are presented as mean ± standard deviation or number (percentage). * *p*-values were derived from univariate analyses; a two-sided *p*-value < 0.05 was considered statistically significant.

**Table 2:** Etiology of liver cirrhosis.

Etiology	No Sepsis (*n* = 130)	Sepsis (*n* = 41)	*p*-Value
Alcohol-related, *n* (%)	20 (15.4)	5 (12.2)	0.802
MASH *	71 (54.6)	23 (56.1)	0.859
Other etiologies ^†^	39 (30.0)	13 (31.7)	0.849

* MASH = metabolic dysfunction-associated steatohepatitis. ^†^ Other etiologies include autoimmune hepatitis, hepatitis B virus (HBV) hepatitis C virus (HCV), autoimmune, primary biliary cholangitis, primary sclerosing cholangitis, and Wilson disease. *p*-value reflects the overall comparison across etiological categories. *p*-values were derived from univariate analyses; a two-sided *p*-value < 0.05 was considered statistically significant.

**Table 3:** Cirrhosis-related complications.

Complication	No Sepsis	Sepsis	*p*-Value
Acute Decompensation	118 (90.7)	40 (97.5)	0.194
Ascites, *n* (%)	34 (26.2)	22 (53.7)	0.001
Hepatic encephalopathy, *n* (%)	33 (25.4)	13 (31.7)	0.545
Esophageal varices, *n* (%)	46 (35.4)	5 (12.2)	0.006
Acute kidney injury, *n* (%)	5 (3.8)	0 (0.0)	0.339
ACLF, *n* (%)	14 (10.8)	20 (48.8)	<0.001

ACLF = acute-on-chronic liver failure. *p*-values were calculated using χ^2^ or Fisher’s exact test, as appropriate. *p*-values < 0.05 were considered statistically significant.

**Table 4:** Liver disease severity scores.

Score	No Sepsis	Sepsis	*p*-Value
MELD-Na, median (IQR)	13.3 (10.0–19.1)	17.8 (13.6–24.7)	<0.001
Child–Pugh score, median (IQR)	7 (6–9)	9 (7.5–10)	<0.001
CLIF-C-ACLF score, median (IQR)	40 (35–45)	45 (37.5–52)	0.006
FIB-4, median (IQR)	4.32 (2.50–8.24)	5.61 (2.91–13.57)	0.054
* CLIF-C-SOFA, median (IQR)	8.00 (7–9)	7 (6–9)	0.106

* Modified CLIF-C-OF without respiratory variables and binary encephalopathy variables. MELD—model for end-stage liver disease [23]. CLIF-C-ACLF: chronic liver failure consortium—acute-on-chronic liver failure [25]. FIB-4 fibrosis-4 index [22]. IQR: interquartile range. Values are presented as medians (interquartile range 25–75%). *p*-values < 0.05 were considered statistically significant.

**Table 5:** (**A**). Hematological parameters. (**B**). Biochemical and coagulation parameters.

**(A)**			
**Parameter**	**No Sepsis**	**Sepsis**	***p*-Value**
Hemoglobin * (g/dL), mean ± SD	10.4 ± 2.5	9.9 ± 2.0	0.29
White blood cells (×10^9^/L), median (IQR)	5.6 (4.0–8.0)	6.6 (4.1–7.6)	0.39
Platelets (×10^9^/L), median (IQR)	119 (85–181)	98 (70–156)	0.33
Neutrophil-to-lymphocyte ratio, median (IQR)	3.0 (2.0–5.3)	5.0 (3.0–8.5)	0.002
(**B**)			
	**No Sepsis**	**Sepsis**	***p*** **-Value**
Creatinine (mg/dL), median (IQR)	0.80 (0.70–1.49)	1.35 (0.80–3.35)	<0.001
Sodium ** (mmol/L), mean ± SD	137.2 ± 4.3	136.9 ± 5.3	0.76
Total bilirubin (mg/dL), median (IQR)	0.90 (0.59–1.70)	1.90 (0.61–2.85)	0.049
INR, median (IQR)	1.30 (1.10–1.50)	1.50 (1.20–1.80)	0.011
CRP (mg/L), median (IQR)	19 (7–47)	33.5 (8–80.8)	0.086
Fibrinogen (mg/dL), median (IQR)	382 (288–460)	343 (220–468)	0.14

* Hemoglobin was normally distributed. Values are presented as mean ± SD or median (IQR). *p*-values < 0.05 were considered statistically significant. ** Sodium was normally distributed. INR = international normalized ratio; values are presented as mean ± SD or median (IQR). CRP = C-Reactive Protein. *p*-values < 0.05 were considered statistically significant.

**Table 6:** Clinical outcomes.

Outcome	No Sepsis (*n* = 130)	Sepsis (*n* = 41)	*p*-Value
Mortality, *n* (%)	16 (12.3)	20 (48.8)	<0.001

*p*-values < 0.05 were considered statistically significant.

**Table 7:** (**A**). Univariable logistic regression analysis for predictors of sepsis. (**B.1**) Multivariable logistic regression analysis for predictors of sepsis (*N* = 171). (**B.2**) Multivariable logistic regression analysis for predictors of sepsis (*N* = 164).

(**A**)				
**Variable**	**Adjusted OR**	**95% CI**	***p*-Value**	
Age (per year)	1.02	0.99–1.05	0.172	
Ethnicity (Jewish vs. Arab)	0.50	0.25–1.02	0.057	
Sex (male vs. female)	0.90	0.44–1.83	0.771	
Ascites	3.23	1.56–6.91	0.002	
Esophageal varices	0.21	0.07–0.57	0.002	
INR	2.06	1.08–3.91	0.027	
Creatinine (mg/dL)	1.76	1.31–2.35	<0.001	
Sodium (mmol/L)	0.99	0.91–1.07	0.758	
Platelet count (×10^9^/L)	1.00	0.96–1.00	0.815	
Fibrinogen (mg/dL)	1.00	0.98–1.00	0.673	
NLR	1.10	1.02–1.19	0.015	
CRP (mg/L)	1.01	1.00–0.01	0.015	
Bilirubin (mg/dL)	1.08	0.97–1.20	0.160	
MELD-Na	1.08	1.03–1.13	<0.001	
CTP	1.42	1.18–1.70	<0.001	
CLIF-C-ACLF	1.05	1.01–1.10	0.008	
(**B.1**)				
**Variable**	**Adjusted OR**	**95% CI**	***p*** **-Value**	**VIF**
Age	1.00	0.97–1.04	0.632	1.06
Ethnicity (Arab)	0.78	0.34–1.78	0.564	1.07
Sex (Male)	0.82	0.36–1.87	0.655	1.04
Ascites	1.58	0.69–4.37	0.235	1.28
Esophageal varices	0.82	0.10–1.15	0.126	1.27
INR	1.27	0.53–3.03	0.594	1.13
Creatinine (mg/dL)	1.58	1.08–2.33	0.019	1.25
NLR	0.993	0.89–1.11	0.900	1.45
CRP (mg/L)	1.01	0.99–1.02	0.090	1.57
(**B.2**)				
**Variable**	**Adjusted OR**	**95% CI**	***p*** **-Value**	**VIF**
Age	1.01	0.98–1.04	0.473	1.05
Ethnicity (Arab)	0.86	0.34–1.78	0.776	1.05
Sex (Male)	0.82	0.36–1.87	0.641	1.03
Ascites	1.74	0.69–4.37	0.235	1.25
Esophageal varices	0.34	0.10–1.15	0.082	1.23
INR	1.14	0.55–2.38	0.719	1.15
Creatinine (mg/dL)	1.55	1.06–2.19	0.010	1.19
NLR	0.94	0.93–1.13	0.585	1.43
CRP (mg/L)	1.00	0.99–1.01	0.617	1.45

Adjusted odds ratios (ORs) were derived from a univariable logistic regression model assessing predictors of sepsis. Continuous variables are expressed per unit increase. OR, odds ratio; CI, confidence interval; INR, international normalized ratio; NLR, neutrophil-to-lymphocyte ratio; CRP, C-reactive protein; MELD, model for end-stage liver disease [23]. CTP, Child–Turcotte–Pugh, CLIF-C-ACLF, chronic liver failure consortium—acute-on-chronic liver failure [25]. *p*-values < 0.05 were considered statistically significant. (B.1) and (B.2): Adjusted odds ratios (ORs) were derived from a multivariable logistic regression model assessing predictors of sepsis. Continuous variables are expressed per unit increase. Multicollinearity among predictors was assessed using variance inflation factors (VIFs); values < 2 indicate no significant collinearity. OR, odds ratio; CI, confidence interval; VIF, variance inflation factor; INR, international normalized ratio; NLR, neutrophil-to-lymphocyte ratio; CRP, C-reactive protein. *p*-values < 0.05 were considered statistically significant.

**Table 8:** Discriminative performance (AUC) of the multivariable model and established liver disease severity scores for prediction of sepsis.

			95% Confidence Interval		
	AUC	Std. Error	Lower	Upper	*p*
predict	0.741	0.0480	0.647	0.835	<0.001
MELD-Na	0.666	0.0483	0.571	0.761	<0.001
child score	0.698	0.0465	0.606	0.789	<0.001
CLIF-C-ACLF	0.637	0.0540	0.532	0.743	0.011
CLIF-SOFA	0.690	0.0506	0.590	0.789	<0.001

Area under the receiver operating characteristic curve (AUC) with 95% confidence intervals is presented for each model. Standard errors were derived from ROC analysis. *p*-values test whether the AUC differs from 0.5 (no discrimination). MELD-Na = Model for End-Stage Liver Disease–Sodium; CLIF-C-ACLF = chronic liver failure consortium—acute-on-chronic liver failure score; CLIF-SOFA = chronic liver failure sequential organ failure assessment; Child score = Child-Pugh Score.

**Table 9:** Calibration of the multivariable prediction model across quartiles of predicted sepsis risk.

Predicted Risk Quartile	*N*	Mean Predicted Probability	Observed Sepsis Rate
Q1 (0.041–0.106)	41	0.070	0.098
Q2 (0.106–0.192)	41	0.148	0.171
Q3 (0.192–0.324)	41	0.254	0.220
Q4 (0.324–0.895)	41	0.504	0.488

Patients were grouped into quartiles based on the predicted probability of sepsis derived from the multivariable logistic regression model. Mean predicted probabilities were compared with observed event rates within each quartile to assess model calibration. Q1: quartile 1, Q2: quartile 2, Q3: quartile 1, Q4: quartile 4. *N* = number of patients = 164.

**Table 10:** Bootstrap internal validation of the multivariable logistic regression model.

Metric	Apparent Performance	Optimism-Corrected Performance
AUC	0.741	0.681
Calibration slope	1.000	0.713

Internal validation was conducted using bootstrap resampling with 5000 iterations. Optimism-corrected performance was calculated by subtracting the mean bootstrap optimism from the apparent model performance. AUC; area under the curve.

**Table 11:** Sepsis incidence according to creatinine categories.

Creatinine Category (mg/dL)	Sepsis (%)
<1.0	17.4%
1.0–1.5	19.2%
1.5–2.0	18.8%
>2.0	48.6%

Creatinine categories were defined as <1.0, 1.0–1.5, 1.5–2.0, and >2.0 mg/dL. Data are presented as counts and percentages. *p*-values were derived from the Chi-square test.

## Data Availability

The data presented in this study are available on request from the corresponding author due to [ethical reasons and Helsinki committee restrictions].
